# Educational Value of a Leadership-Focused High-Fidelity Simulation Scenario in Undergraduate Nursing Education: A Single-Group Educational Intervention Study

**DOI:** 10.3390/nursrep16080271

**Published:** 2026-08-04

**Authors:** Chennyfer Dobbins Abi Rached, Ana Luiza Oliveira Sinzato, Thacyane Samara dos Santos, Victoria Dorneles Nery Galiotti, Reginaldo Aguiar, Irene Mari Pereira, Nertila Podgorica

**Affiliations:** 1 School of Nursing, University of São Paulo, 419, Av. Dr. Enéas Carvalho de Aguiar, Cerqueira César, São Paulo 05403-000, SP, Brazilvictoria.galiotti@usp.br (V.D.N.G.);; 2 Central Institute, Hospital das Clínicas, Faculty of Medicine, University of São Paulo (ICHC-HCFMUSP), R. 225 Dr. Ovídio Pires de Campos, Cerqueira César, São Paulo 05403-010, SP, Brazil; 3 Department of Nursing Science and Gerontology, Institute of Nursing Science, UMIT TIROL—Private University for Health Sciences and Health Technology, 6060 Hall in Tirol, Austria; nertila.podgorica@umit-tirol.at

**Keywords:** leadership, nursing education, debriefing, student perceptions, simulation-based learning, needlestick injuries

## Abstract

**Aim/Objective:** To evaluate undergraduate nursing students’ perceptions of the educational design, debriefing quality, satisfaction, and self-confidence associated with a leadership-focused high-fidelity simulation scenario involving the management of a sharps-injury event. **Background:** High-fidelity simulation provides structured opportunities for undergraduate nursing students to support learning of communication, prioritization, decision-making, teamwork, and leadership-related behaviors. **Design:** Single-group educational intervention study using post-intervention learner-reported outcomes. **Methods:** Fourth-year undergraduate nursing students from a public university in Brazil participated in a 15-min leadership-focused high-fidelity simulation involving the management of a sharps-injury event. After the simulation, participants completed validated instruments assessing simulation design, satisfaction and self-confidence in learning, educational practices, and debriefing. Descriptive statistics and exploratory Pearson correlation analyses were performed using complete data from all participants. **Results:** Fifty-six students participated in the simulation. Overall, participants reported favorable perceptions of the simulation design, satisfaction with learning, self-confidence, educational practices, and debriefing. Exploratory analyses showed positive correlations between satisfaction and active learning, high expectations, and debriefing quality, as well as between self-confidence and active learning, debriefing, and high expectations. **Conclusions:** Undergraduate nursing students perceived the leadership-focused high-fidelity simulation scenario as realistic, reflective, and educationally valuable. The scenario may represent a useful pedagogical strategy for introducing leadership-related behaviors in undergraduate nursing education. However, because the findings were based on self-reported perceptions collected after a single simulation activity, they should not be interpreted as evidence of objective leadership-related behaviors or transfer of learning to clinical practice.

## 1. Introduction

Healthcare simulation is a standards-based educational strategy that recreates clinical situations for learning, reflection, assessment, and improvement. Its educational quality depends on intentional design, clear objectives, facilitation, prebriefing, debriefing, and adherence to best-practice standards [1,2,3,4,5].

Simulation-based education is widely used to support clinical reasoning, decision-making, and the integration of theoretical knowledge with practice in nursing education [6,7,8]. Beyond technical skills, simulation can foster communication, teamwork, prioritization, problem-solving, emotional support, and leadership-related behaviors that are essential for care coordination, interprofessional collaboration, and patient safety [9,10,11]. Because simulation allows learners to respond to evolving clinical situations and reflect on their actions during debriefing, it has been recognized as a valuable strategy for leadership-focused education in healthcare education [10,12].

Despite growing interest in leadership-focused simulation, evidence describing undergraduate nursing students’ educational experiences with leadership-focused simulation remains limited [13]. In this context, perception-based outcomes may provide useful information about how learners experience simulation design, realism, feedback, debriefing, and educational practices, all of which have been associated with satisfaction and self-confidence in simulation-based learning [14].

Although simulation has been increasingly used in nursing education, less is known about how undergraduate students perceive leadership-oriented simulation scenarios that address nontechnical competencies such as communication, prioritization, decision-making, emotional support, and team coordination. In particular, sharps-injury management represents a clinically relevant situation in which leadership-related behaviors may be introduced in a safe educational environment. Given the exploratory nature of this study, no a priori hypotheses were formulated. This study therefore aimed to evaluate undergraduate nursing students’ perceptions of the educational design, debriefing quality, satisfaction, and self-confidence associated with a leadership-focused high-fidelity simulation scenario involving the management of a sharps-injury event. The study also explored associations among simulation design, educational practices, satisfaction, self-confidence, and debriefing domains.

## 2. Materials and Methods

### 2.1. Study Design, Reporting Standards, and Setting

This quantitative single-group educational intervention study evaluated undergraduate nursing students’ perceptions following participation in a leadership-focused high-fidelity simulation. The study employed a post-intervention assessment using validated learner-reported outcome measures. Because the educational intervention was implemented as part of a regular undergraduate course, no comparison group was included. The manuscript was prepared according to the TREND Statement and the Reporting Guidelines for Healthcare Simulation Research [15,16]. The simulation activity was designed in accordance with the Healthcare Simulation Standards of Best Practice ^®^ [1,2,3,4,5,17].

### 2.2. Participants, Recruitment, Eligibility, and Analytic Sample

The study used a convenience sample of fourth-year undergraduate nursing students enrolled in a leadership-related course at a public university in southeastern Brazil. All students enrolled in the course during the study period were invited to participate. Recruitment took place during regular course activities, and no additional advertising or external recruitment strategies were used. Participation in the educational intervention formed part of the curriculum, whereas participation in the research component, including completion and use of the post-intervention questionnaires, was entirely voluntary.

All participants were fourth-year (eighth-semester) undergraduate nursing students enrolled in the same management class and had no previous experience with high-fidelity simulation before participating in the educational intervention.

Eligible participants attended the simulation activity, participated in the structured debriefing session, and provided written informed consent before completing the study instruments. Complete questionnaire data were obtained from all 56 participants; therefore, the final analytic sample included the entire cohort, with no exclusions or missing data. Because this educational intervention was embedded within a mandatory undergraduate course, the available sample comprised the entire cohort of fourth-year nursing students enrolled during the study period. Consequently, no a priori sample size calculation was performed. The study should therefore be considered exploratory and hypothesis-generating rather than confirmatory.

Descriptive analyses, domain-level scores, and exploratory Pearson correlation analyses were conducted using complete-case data from all participants. Participant flow and analytic denominators are summarized in Table 1, where sample sizes, confidence intervals, and statistical results are also presented.

### 2.3. Simulation Scenario and Educational Intervention

Data were collected following a leadership-focused high-fidelity simulation scenario entitled “Accident With a Sharp Object,” which was designed to promote communication, prioritization, decision-making, teamwork, emotional support, and leadership-related behaviors. The scenario was developed using principles of the Jeffries Simulation Framework and collaborative leadership concepts [18,19]. A high-fidelity adult patient simulator (HAL S3201, Gaumard Scientific, Miami, FL, USA) and trained actors were used. The scenario underwent faculty review and pilot testing before implementation.

The educational intervention was standardized for all participants. All simulation sessions followed the same learning objectives, prebriefing process, scenario script, facilitator guidance, and structured debriefing format. Sessions were conducted by faculty members with previous experience in high-fidelity simulation and undergraduate nursing education who had received institutional training in simulation facilitation and debriefing. Standardized facilitator guidance and identical prebriefing, scenario, and debriefing procedures were used to ensure consistency across all sessions.

During the simulation, facilitators deliberately promoted participant autonomy and did not provide direct guidance regarding clinical or leadership decisions. Their role was limited to maintaining scenario fidelity and ensuring progression according to the predefined script while preserving participant safety. Students were encouraged to independently prioritize actions, communicate with team members, and make clinical and leadership decisions throughout the scenario. Facilitators intervened only when necessary to maintain scenario continuity, ensure participant safety, or address technical issues.

### 2.4. Session Structure and Simulation Procedures

Each session included prebriefing, scenario enactment, and structured debriefing. Students either participated directly in the scenario or served as observers using a structured observation guide. Following the debriefing session, participants completed the study instruments (Table 2).

### 2.5. Instruments

Students completed four validated self-report instruments: the Simulation Design Scale (SDS), the Student Satisfaction and Self-Confidence in Learning Scale (SSSCL), the Educational Practices Questionnaire (EPQ), and the Debriefing Assessment for Simulation in Healthcare—Student Version, Short Form (DASH-SV Short). Portuguese-language versions of these instruments have been translated, culturally adapted, and validated for use in simulation-based education contexts [20,21,22,23]. Higher scores indicated more favorable perceptions of simulation design, educational practices, satisfaction, self-confidence, and debriefing quality.

The Portuguese-language versions of the Simulation Design Scale (SDS), Student Satisfaction and Self-Confidence in Learning Scale (SSSCL), Educational Practices Questionnaire (EPQ), and Debriefing Assessment for Simulation in Healthcare–Student Version, Short Form (DASH-SV Short) have been translated, culturally adapted, and validated for use in simulation-based education contexts. In the present sample, the instruments demonstrated good to excellent internal consistency, with Cronbach’s alpha coefficients of 0.95 for the SDS, 0.91 for the SSSCL, 0.89 for the EPQ, and 0.94 for the DASH-SV Short.

### 2.6. Data Collection Procedures

Data were collected immediately after completion of the simulation and debriefing activities. Participation was voluntary, and questionnaire responses were completed anonymously. Data were analyzed using IBM SPSS Statistics version 25.0 [24]. Complete data were available for all participants; therefore, no missing-data procedures were required.

### 2.7. Statistical Analysis

Descriptive statistics were calculated for each instrument item and domain, including the number of valid responses, missing values, means, standard deviations, minimum and maximum values, quartiles, medians, and 95% confidence intervals where appropriate. Because the primary aim was exploratory, descriptive results were used to characterize students’ perceptions of simulation design, satisfaction, self-confidence, educational practices, and debriefing.

Prior to correlation analyses, distributions were inspected descriptively. Pearson correlations were selected because domain scores were treated as approximately continuous composite measures and sample size was considered adequate for exploratory correlation analyses.

Correlation analyses were prespecified as exploratory. Therefore, *p*-values were adjusted using the Benjamini–Hochberg false discovery rate (FDR) procedure to account for multiple comparisons while controlling the expected proportion of false-positive findings. The complete exploratory correlation matrix is provided in Supplementary File S2 for transparency and descriptive purposes and was not used for confirmatory inference.

Correlation magnitudes were interpreted using Cohen’s conventional benchmarks: approximately r = 0.10 as small, r = 0.30 as medium, and r ≥ 0.50 as large [25]. However, very large correlations were not overinterpreted because they may reflect overlapping constructs, ceiling effects, common-method variance, or measurement dependencies among closely related domains.

All instruments demonstrated good to excellent internal consistency (SDS α = 0.95, SSSCL α = 0.91, EPQ α = 0.89, and DASH-SV Short α = 0.94).

### 2.8. Ethical Considerations

The study followed institutional ethical requirements and was approved by the Research Ethics Committee of the School of Nursing, University of São Paulo (CEP/EEUSP), under CAAE no. 85072824.8.0000.5392, technical opinion no. 7.455.186, approved in March 2025. Students received information about the study aims, procedures, voluntary nature of participation, anonymous questionnaire completion, and absence of academic penalty for refusal or nonparticipation. Questionnaires were completed anonymously, and faculty responsible for course grading had no access to individual responses until after completion of academic evaluation. Participation or nonparticipation in the study had no influence on course grades or academic assessment. Students attended the simulation as part of the regular undergraduate curriculum. Participation in the research component was voluntary and referred exclusively to completion and use of anonymous questionnaire data for research purposes. Students were informed that refusal to participate in the study would not affect their course participation, academic assessment, or grades.

## 3. Results

### 3.1. Participant Flow and Analytic Sample

All fourth-year undergraduate nursing students enrolled in the leadership course during the study period were invited to participate in the educational intervention. Fifty-six students attended the simulation activity, participated in the structured debriefing session, and subsequently completed the post-intervention questionnaires. Consequently, no participants were excluded from the analyses, no questionnaires contained missing data, and complete-case analysis was performed. Participants had a mean age of 22.71 years (SD = 1.16); 51 (91.1%) were female and 5 (8.9%) were male. All participants were fourth-year (eighth-semester) undergraduate nursing students enrolled in the same leadership course, and none had previous experience with high-fidelity simulation. Detailed descriptive statistics and the complete exploratory correlation matrix are presented in Supplementary Files S1 and S2.

All simulation sessions were completed as planned. No adverse events, participant injuries, psychological distress requiring intervention, or interruptions to the educational activities were reported during the study. No unintended effects related to the educational intervention were identified.

### 3.2. Descriptive Results

Students reported generally favorable perceptions across all study domains (Table 3). Among the SDS domains, feedback/reflection, realism, and support received the highest ratings, whereas objectives/information received the comparatively lowest rating. Debriefing evaluations were highly positive, with high scores observed for both the introduction element and the overall debriefing composite.

### 3.3. Exploratory Correlation Analysis

Exploratory Pearson correlations identified several positive associations. Statistical significance for the prespecified correlations reported in Table 4 was evaluated using Benjamini–Hochberg false discovery rate-adjusted *p*-values. Satisfaction with learning was positively associated with active learning (r = 0.355, *p* = 0.007) and high expectations (r = 0.329, *p* = 0.013). Self-confidence in learning showed positive associations with the debriefing introduction element (r = 0.307, *p* = 0.021), overall debriefing quality (r = 0.303, *p* = 0.023), active learning (r = 0.354, *p* = 0.008), and high expectations (r = 0.268, *p* = 0.046).

Among simulation-design and debriefing variables, objectives/information demonstrated a positive association with debriefing quality (r = 0.264, *p* = 0.049). A moderate positive association was also observed between the debriefing introduction element and overall debriefing quality (r = 0.527, *p* < 0.001). The complete exploratory correlation matrix is presented in Supplementary File S2.

## 4. Discussion

This exploratory study examined undergraduate nursing students’ perceptions of the educational experience following a leadership-focused high-fidelity simulation. Students reported favorable perceptions across all evaluated domains, particularly debriefing, realism, active learning, diverse learning opportunities, and self-confidence in learning. These findings suggest that students perceived the simulation as educationally valuable, although the results should be interpreted as perceptions of the learning experience rather than evidence of objective leadership-related behaviors or transfer to clinical practice [10,15,16]. Positive ratings for prebriefing, objectives, realism, and debriefing are consistent with contemporary healthcare simulation standards [1,3].

The favorable learner-reported outcomes observed in the present study are also consistent with recent international evidence. Bdiri Gabbouj et al. [14], using the same educational instruments (SDS, SSSCL, and EPQ), reported mean scores equivalent to approximately 4.20/5 for satisfaction and 4.23/5 for self-confidence. In comparison, our participants reported mean scores of 4.55 and 4.34, respectively. Although both studies indicate highly positive learner perceptions, the slightly higher ratings observed in the present study may reflect differences in participants’ academic level, the leadership-focused scenario, facilitation procedures, or previous simulation experience.

A relevant contribution of this study is the use of a sharps-injury scenario as the context for developing clinical leadership behaviors. Unlike simulation scenarios centered primarily on advanced life support algorithms or technical procedures, this scenario required students to respond to an unexpected occupational exposure involving a team member. Participants were challenged to prioritize actions, coordinate the team, communicate effectively, provide emotional support to an injured colleague, and maintain continuity of patient care. These demands placed leadership within an emotionally demanding and realistic clinical context in which students were required to respond not only to the technical aspects of occupational exposure but also to the uncertainty, emotional distress, and workflow disruption associated with an unexpected adverse event. Managing this situation required rapid prioritization, clear communication, team coordination, emotional support for an injured colleague, and maintenance of patient care, reinforcing leadership as a clinical competence rather than solely a managerial role. This interpretation is consistent with previous reviews highlighting simulation as an effective strategy for developing leadership competencies in undergraduate nursing education [12].

The positive associations involving prebriefing, objectives, and debriefing suggest that students may engage more effectively in reflective learning when expectations, roles, and learning goals are clearly established. This relationship is pedagogically plausible because effective prebriefing promotes psychological safety and learner readiness, whereas debriefing supports reflection on communication, prioritization, teamwork, and decision-making [3].

The findings support the inclusion of leadership-focused education during undergraduate nursing education. Nursing leadership extends beyond formal managerial roles and includes communication, prioritization, delegation, care coordination, situational awareness, and decision-making in complex clinical environments [9,11,13]. These competencies are associated with patient safety, quality of care, and team performance.

The favorable ratings for realism and problem-solving suggest that students perceived the sharps-injury scenario as authentic and educationally challenging. However, these perceptions should not be interpreted as evidence that leadership-related behaviors were objectively acquired or demonstrated.

The exploratory correlations should be interpreted as descriptive indicators of how students perceived different components of the simulation experience rather than as evidence of causal relationships. Because all variables were measured through self-report instruments at a single time point, the observed associations may partly reflect overlapping constructs, common-method variance, or generally favorable learner evaluations of the activity.

Although statistical significance was evaluated after controlling the false discovery rate for the prespecified correlations, these associations remain exploratory and should not be interpreted as evidence of causal relationships.

Our correlation findings are also broadly consistent with previous international evidence. Bdiri Gabbouj et al. [14] demonstrated positive associations between simulation-design characteristics, educational practices, satisfaction, and self-confidence. In their study, the support domain showed moderate correlations with satisfaction (r = 0.468) and self-confidence (r = 0.477), whereas diverse ways of learning demonstrated even stronger associations with learner outcomes. Similarly, satisfaction in the present study was positively associated with active learning (r = 0.355), suggesting that students who perceived greater engagement in the educational process also reported more favorable learning experiences. Conversely, self-confidence was not significantly associated with several Simulation Design Scale domains, including support, problem-solving, feedback/reflection, and realism. One possible interpretation is that favorable perceptions of simulation design alone may be insufficient to enhance learners’ confidence without the active engagement and structured reflection promoted through educational practices and debriefing. Despite differences in the magnitude of these associations, both studies support the importance of educational design and learner engagement in maximizing the perceived benefits of simulation [14].

Despite receiving the lowest SDS score, ratings for the Support domain remained favorable. One possible, untested explanation is that leadership-focused scenarios may encourage learner autonomy and decision-making under uncertainty, which can reduce perceptions of facilitator support despite appropriate instructional design. This hypothesis may also be consistent with the facilitation strategy adopted in the present study, which intentionally emphasized learner autonomy while limiting facilitator intervention during scenario enactment. Future studies should further explore the balance between learner autonomy and facilitator guidance in leadership-focused simulation.

Debriefing emerged as one of the strongest components of the learning experience and showed a positive association with self-confidence in learning. This finding aligns with simulation standards and the Debriefing for Meaningful Learning framework, which identify debriefing as a central mechanism for reflection, clinical reasoning, and knowledge integration [1,26].

In leadership-focused simulation, debriefing allows learners to critically examine communication, delegation, prioritization, emotional responses, and team interactions that may not be fully recognized during scenario enactment. Consistent with this interpretation, the favorable ratings observed across all DASH-SV Short elements reinforce the role of structured debriefing as a key contributor to meaningful learning, knowledge integration, and professional development, supporting contemporary simulation standards that position debriefing as one of the most influential mechanisms through which learning is consolidated and meaning is constructed [1,27,28].

Our findings also complement those reported by Rached et al. [29], who described favorable student perceptions following an escape-room strategy designed to promote leadership competencies. Although different educational approaches were employed, both studies suggest that active learning environments foster communication, collaboration, prioritization, and decision-making among undergraduate nursing students. The present study extends this evidence by embedding these competencies within a high-fidelity simulation involving an unexpected occupational exposure, thereby reproducing a realistic clinical situation that requires simultaneous technical and non-technical responses. Collectively, these findings reinforce previous recommendations that simulation-based education should provide authentic opportunities for learners to develop leadership competencies in complex clinical environments [10,12,29].

Overall, the findings are consistent with a growing body of international evidence supporting high-fidelity simulation as an effective educational strategy in undergraduate nursing education. Recent international studies have reported favorable educational outcomes following simulation-based learning, including high levels of learner satisfaction, self-confidence, engagement, and perceived competence [14,30,31]. Although differences in study design, participant characteristics, educational settings, and simulation scenarios preclude direct comparison of absolute scores, the convergence of these findings strengthens the interpretation that carefully designed simulation experiences consistently promote positive learner perceptions across diverse educational contexts.

An additional contribution of this study lies in the educational context selected for leadership development. Unlike the resuscitation-based or procedure-focused simulation scenarios more frequently reported in the literature, the sharps-injury scenario required students to integrate communication, prioritization, delegation, teamwork, emotional support, and clinical decision-making while managing an unexpected occupational exposure under conditions of uncertainty and emotional pressure. This approach situates leadership within a realistic and psychologically challenging clinical situation that undergraduate nursing students are likely to encounter in practice, reinforcing leadership as a set of observable clinical behaviors rather than solely a managerial function. Future multicenter studies should combine subjective measures with objective assessments of leadership performance, behavioral observation, and longitudinal follow-up to determine whether these favorable learner perceptions translate into sustained behavioral change and improved clinical practice [14,30,31].

Collectively, these findings suggest that leadership-focused high-fidelity simulation, when supported by structured preparation and high-quality debriefing, provides an educational environment that students perceive as realistic, reflective, engaging, and conducive to leadership learning.

### 4.1. Implications for Nursing Education and Simulation Design

The findings have several practical implications for simulation educators. First, leadership-focused simulation objectives should be written in observable behavioral terms rather than as broad conceptual aims. Instead of stating only that the goal is leadership-focused education, objectives should specify behaviors such as identifying priorities, initiating communication, delegating tasks, supporting an affected team member, escalating concerns, documenting actions, and reflecting on team performance. This approach aligns with the outcomes-and-objectives standard, which emphasizes that simulation-based experiences should be linked to measurable learning outcomes [17].

Second, prebriefing should be treated as a substantive educational phase rather than as a brief procedural introduction. Although support received comparatively lower ratings than the other simulation-design domains, scores remained favorable overall. Future iterations of the scenario should strengthen orientation to learning objectives, participant roles, observer responsibilities, confidentiality, psychological safety, and the leadership focus of the scenario [3,5].

Third, debriefing should be protected as a core instructional component. The associations between debriefing, satisfaction, and self-confidence suggest that reducing debriefing time may compromise the educational value of leadership-focused simulation. Facilitators should be trained to guide reflection not only on clinical actions but also on communication, prioritization, emotional responses, team coordination, and decision-making [1,26].

Fourth, leadership-focused simulation should be embedded longitudinally across the undergraduate curriculum. A single simulation session may introduce students to leadership-related learning experiences, but competence development requires repeated exposure to progressively complex situations. Costa et al. [13] emphasized that leadership-focused education in undergraduate nursing should be structured, continuous, and integrated across curricula. A progressive simulation sequence could begin with communication and recognition of risk, then advance to delegation, interprofessional conflict, competing priorities, and crisis coordination. This approach would allow students to revisit leadership behaviors over time and receive repeated feedback.

### 4.2. Strengths and Limitations

The study was conducted in a single educational setting and involved participants from a single undergraduate nursing program, which may limit the generalizability of the findings to other institutions and educational contexts. Nevertheless, it addresses leadership learning, an important but still relatively underexplored area in undergraduate simulation-based nursing education. A further strength is the inclusion of the entire cohort of students participating in the educational activity, resulting in a complete analytic sample of 56 participants with no missing questionnaire data.

The study also examined multiple process-oriented domains rather than relying solely on satisfaction or self-confidence outcomes. By simultaneously assessing simulation design, educational practices, debriefing quality, satisfaction, and self-confidence, the study provides a broader understanding of how students perceived the educational structure of the simulation experience. In addition, the scenario was clinically meaningful and explicitly focused on leadership-related competencies, and the interpretation of the findings was grounded in contemporary healthcare simulation standards and best-practice recommendations.

Several limitations should be considered. First, the intervention was implemented as part of a regular undergraduate curricular activity; therefore, no comparison group was included. Although the study provides valuable information regarding students’ perceptions of the educational intervention, it does not permit causal inference regarding its effectiveness or allow attribution of the observed outcomes solely to the intervention. Furthermore, the study relied exclusively on self-reported perceptions collected immediately after the simulation activity. Accordingly, the findings reflect students’ subjective evaluations of the educational experience rather than objective evidence of leadership-related behaviors, knowledge acquisition, or transfer of learning to clinical practice. Because the questionnaires were completed anonymously but within the educational setting immediately after the activity, responses may have been influenced by social desirability. Objective assessments of leadership performance, behavioral observation, and longitudinal evaluation of skill transfer into clinical practice were beyond the scope of the present study. Consequently, the absence of a pre-intervention assessment, objective performance measures, and longitudinal follow-up limits conclusions regarding educational outcomes. Finally, although the sample included the entire cohort of students enrolled in the course, it was drawn from a single institution and a single academic cohort, which may limit the generalizability of the findings.

An additional limitation of this study is the absence of a comparator group. Without a control or comparison condition, it is not possible to determine whether the favorable perceptions observed were attributable specifically to the leadership-focused simulation, to simulation-based education more generally, or to other contextual aspects of the educational experience. Consequently, causal inferences regarding the effectiveness of the intervention cannot be drawn. Future controlled or randomized studies comparing leadership-focused simulation with alternative educational strategies are warranted to provide more robust evidence of its educational impact.

### 4.3. Future Research

Future studies should build on these findings using stronger designs and more diverse samples. Multi-site studies would improve generalizability and precision. Longitudinal studies would help determine whether repeated leadership-focused simulations influence students’ self-confidence, observable leadership behaviors, and transfer to clinical placements over time. Mixed-methods studies would also be valuable because interviews, focus groups, or analysis of debriefing transcripts could clarify how students interpret leadership, what they find difficult about exercising it, and which elements of simulation design they perceive as most supportive.

Future research should also move beyond self-report outcomes. Satisfaction and self-confidence are useful learner-reported indicators, but they are insufficient as stand-alone evidence of leadership-related behaviors. Subsequent studies should incorporate structured observer checklists, faculty-rated leadership rubrics, peer assessment, standardized behavioral indicators, or video-assisted analysis of communication, delegation, and decision-making. Such approaches would align leadership-focused simulation research with calls for stronger evidence of performance and transfer in healthcare simulation [10,15,17].

## 5. Conclusions

This exploratory study found that undergraduate nursing students perceived a leadership-focused high-fidelity simulation scenario as realistic, reflective, and educationally valuable. The findings suggest that a high-fidelity simulation scenario based on sharps-injury management may provide a useful educational context for introducing leadership-related behaviors, including communication, prioritization, emotional support, team coordination, and decision-making. However, because the study relied on self-reported perceptions collected after a single simulation activity, the results should not be interpreted as evidence of objective leadership-related behaviors, behavioral performance, or transfer of learning to clinical practice. Future studies should incorporate objective performance measures, longitudinal follow-up, and multi-site designs to further examine the contribution of leadership-focused simulation to undergraduate nursing education.

## Figures and Tables

**Table 1:** Participant Flow and Analytic Denominators.

Participant Stage or Analytic Subset	N	Reporting Approach
Students attending the simulation activity	56	Course-based participating cohort
Students with complete post-simulation questionnaire data	56	Principal analytic sample
SSSCL domain analyses	56	Satisfaction and self-confidence domains analyzed using complete cases
SDS domain analyses	56	All SDS domains analyzed using complete cases
DASH-SV Short assessment domains	56	Individual elements and composite scores analyzed using complete cases
EPQ domain analyses	56	Active learning, collaboration, diverse ways of learning, and high expectations domains analyzed using complete cases
Pearson correlation analyses	56 paired observations	Pearson’s r, 95% CI, and *p* values reported

Note. SSSCL = Student Satisfaction and Self-Confidence in Learning Scale; SDS = Simulation Design Scale; EPQ = Educational Practices Questionnaire; DASH-SV Short = Debriefing Assessment for Simulation in Healthcare—Student Version, Short Form.

**Table 2:** Simulation Session Timeline.

Phase	Duration	Purpose
Prebriefing and scenario familiarization	5 min	Orientation to the simulation environment, roles, expectations, and observation focus
Scenario enactment	15 min	Leadership-focused response to a sharps-injury event in a hospital setting
Structured debriefing	30 min	Guided reflection on communication, decision-making, problem-solving, emotional support, and leadership behaviors

**Table 3:** Domain-Level Descriptive Statistics for Simulation, Satisfaction, Self-Confidence, Debriefing, and Educational-Practice Outcomes.

Domain	N	M	SD	95% CI	Scale Range
SSSCL satisfaction with learning	56	4.55	0.45	[4.42, 4.65]	1–5
SSSCL self-confidence in learning	56	4.34	0.49	[4.20, 4.46]	1–5
SDS objectives/information	56	4.26	0.70	[4.06, 4.42]	1–5
SDS support	56	4.71	0.44	[4.57, 4.80]	1–5
SDS problem-solving	56	4.67	0.37	[4.57, 4.76]	1–5
SDS feedback/reflection	56	4.76	0.44	[4.62, 4.85]	1–5
SDS realism	56	4.72	0.52	[4.55, 4.83]	1–5
DASH-SV Short introduction element	56	6.75	0.61	[6.55, 6.88]	1–7
DASH-SV Short debriefing composite	56	6.52	0.59	[6.35, 6.66]	1–7
EPQ active learning	56	4.67	0.28	[4.59, 4.74]	1–5
EPQ collaboration	56	4.86	0.52	[4.66, 4.96]	1–5
EPQ diverse ways of learning	56	4.65	0.40	[4.54, 4.75]	1–5
EPQ high expectations	56	4.78	0.41	[4.65, 4.87]	1–5

Note. SSSCL = Student Satisfaction and Self-Confidence in Learning Scale; SDS = Simulation Design Scale; DASH-SV Short = Debriefing Assessment for Simulation in Healthcare—Student Version, Short Form; EPQ = Educational Practices Questionnaire; N = sample size; M = mean; SD = standard deviation; CI = confidence interval.

**Table 4:** Selected Exploratory Correlations Among Simulation-Design, Debriefing, Satisfaction, and Self-Confidence Domains.

Variable A	Variable B	N	df	r	95% CI	*p*	FDR-Adjusted *p*
Satisfaction with learning	Active learning	56	54	0.355	[0.102, 0.565]	0.007	0.021
Satisfaction with learning	High expectations	56	54	0.329	[0.072, 0.545]	0.013	0.026
Self-confidence in learning	Introduction element	56	54	0.307	[0.048, 0.527]	0.021	0.031
Self-confidence in learning	Debriefing	56	54	0.303	[0.043, 0.524]	0.023	0.031
Self-confidence in learning	Active learning	56	54	0.354	[0.100, 0.564]	0.008	0.021
Self-confidence in learning	High expectations	56	54	0.268	[0.005, 0.496]	0.046	0.049
Objectives/information	Debriefing	56	54	0.264	[0.001, 0.493]	0.049	0.049
Introduction element	Debriefing	56	54	0.527	[0.307, 0.694]	<0.001	0.008

Note. N = sample size; df = degrees of freedom; CI = confidence interval; FDR = false discovery rate. *p*-values were adjusted for multiple comparisons using the Benjamini–Hochberg FDR procedure.

## Data Availability

The data supporting the findings of this study are summarized in the manuscript and Supplementary Materials. Additional deidentified data may be made available by the corresponding author upon reasonable request, subject to institutional and ethical restrictions.

## Supplementary Materials

The following supporting information can be downloaded at: https://www.mdpi.com/article/10.3390/nursrep16080271/s1, Supplementary File S1: Full Item-Level Descriptive Statistics. Supplementary File S2: Full Pairwise Correlation Matrix. Supplementary File S3: TREND Checklist. Supplementary File S4: Simulation Scenario Script and Observer Checklist. Supplementary File S5: Instrument, Scoring, and Permission Documentation Notes [32,33,34].

## Public Involvement Statement

No public involvement in any aspect of this research.

## Guidelines and Standards Statement

This manuscript was prepared in accordance with the Transparent Reporting of Evaluations with Nonrandomized Designs (TREND) Statement for reporting nonrandomized educational intervention studies and the Reporting Guidelines for Healthcare Simulation Research, as applicable.
