# Supplementary material for: Educational Value of a Leadership-Focused High-Fidelity Simulation Scenario in Undergraduate Nursing Education: A Single-Group Educational Intervention Study
Source: Nurs Rep. 2026 Aug 4;16(8):271. doi: 10.3390/nursrep16080271 (PMC13516673; doi:10.3390/nursrep16080271)
Supplement: Supplementary file 1 [file nursrep-16-00271-s001.zip › nursrep-4440907-supplementary.pdf]

Supplementary Materials

Nursing Students' Perceptions of a Leadership-Focused High-Fidelity Simulation Experience: A Cross-Sectional Exploratory Study

Supplementary S1. Full Item-Level Descriptive Statistics

This appendix provides the full item-level and domain-level descriptive statistics that support the domain summaries reported in the main manuscript. Labels have been standardized to match the manuscript. DASH-SV Short labels are used for the debriefing assessment in this submission package.

Note. N = number of valid responses; Missing = number of missing item/domain responses; M = mean; SD = standard deviation; Q1 = first quartile; Q3 = third quartile; CI = confidence interval. Confidence intervals are shown as provided in the analytic output and may slightly exceed theoretical scale limits because they are unbounded normal-theory intervals.

Table S1. Full item-level and domain-level descriptive statistics

Variable	N	Missing	M	SD	Min	Q1	Median	Q3	Max	Lower 95% CI	Upper 95% CI
Age	56	0	22.71	1.16	21	22	23	24	25	22.42	23.02
SSSCL1	56	0	4.75	0.58	3	5	5	5	5	4.56	4.87
SSSCL2	56	0	4.20	0.84	3	3	4	5	5	3.97	4.41
SSSCL3	56	0	4.82	0.51	3	5	5	5	5	4.64	4.92
SSSCL4	56	0	4.43	0.85	2	4	5	5	5	4.18	4.63
SSSCL5	56	0	4.79	0.53	3	5	5	5	5	4.61	4.90
SSSCL6	56	0	4.11	0.95	2	3.75	4	5	5	3.84	4.34
SSSCL7	56	0	4.14	0.86	2	3	4	5	5	3.91	4.36
SSSCL8	56	0	4.46	0.81	2	4	5	5	5	4.22	4.65
SSSCL9	56	0	4.57	0.81	1	4	5	5	5	4.31	4.74
SSSCL10	56	0	4.39	1.02	1	4	5	5	5	4.08	4.62
SSSCL11	56	0	4.48	0.95	1	4	5	5	5	4.17	4.69
SSSCL12	56	0	4.73	0.49	3	4.75	5	5	5	4.59	4.84
SSSCL13	56	0	3.79	1.00	2	3	4	5	5	3.53	4.05
SSSCL satisfaction with learning	56	0	4.55	0.45	3.25	4.25	4.75	5	5	4.42	4.65
SSSCL self-confidence in learning	56	0	4.34	0.49	2.875	4.125	4.312	4.625	5	4.20	4.46
SDS1	56	0	4.32	0.77	3	4	4.5	5	5	4.11	4.51
SDS2	56	0	4.63	0.49	4	4	5	5	5	4.49	4.75
SDS3	56	0	4.11	1.06	2	4	4	5	5	3.81	4.36
SDS4	56	0	3.98	0.92	2	3.75	4	5	5	3.73	4.21
SDS5	56	0	4.25	0.92	2	4	4	5	5	3.98	4.46
SDS6	56	0	4.68	0.72	3	5	5	5	5	4.45	4.84
SDS7	56	0	4.73	0.73	2	5	5	5	5	4.47	4.88
SDS8	56	0	4.52	0.50	4	4	5	5	5	4.39	4.65
SDS9	56	0	4.91	0.29	4	5	5	5	5	4.81	4.97
SDS10	56	0	4.66	0.48	4	4	5	5	5	4.53	4.78
SDS11	56	0	4.68	0.54	3	4	5	5	5	4.52	4.80

Variable	N	Missing	M	SD	Min	Q1	Median	Q3	Max	Lower 95% CI	Upper 95% CI
SDS12	56	0	4.63	0.49	4	4	5	5	5	4.49	4.75
SDS13	56	0	4.75	0.44	4	4.75	5	5	5	4.62	4.85
SDS14	56	0	4.64	0.48	4	4	5	5	5	4.51	4.76
SDS15	56	0	4.71	0.62	3	5	5	5	5	4.52	4.85
SDS16	56	0	4.54	0.71	3	4	5	5	5	4.33	4.70
SDS17	56	0	4.88	0.33	4	5	5	5	5	4.77	4.95
SDS18	56	0	4.91	0.29	4	5	5	5	5	4.81	4.97
SDS19	56	0	4.64	0.59	3	4	5	5	5	4.47	4.78
SDS20	56	0	4.80	0.52	3	5	5	5	5	4.63	4.91
SDS objectives/information	56	0	4.26	0.70	2.8	3.8	4.6	4.8	5	4.06	4.42
SDS support	56	0	4.71	0.44	3.25	4.75	4.75	5	5	4.57	4.80
SDS problem-solving	56	0	4.67	0.37	4	4.35	4.8	5	5	4.57	4.76
SDS feedback/reflection	56	0	4.76	0.44	3.5	4.75	5	5	5	4.62	4.85
SDS realism	56	0	4.72	0.52	3	4.5	5	5	5	4.55	4.83
DASH-SV Short Element 1 (introduction)	56	0	6.75	0.61	5	7	7	7	7	6.55	6.88
DASH-SV Short Element 2 (debriefing)	56	0	6.75	0.58	5	7	7	7	7	6.56	6.87
DASH-SV Short Element 3 (debriefing)	56	0	6.50	0.85	4	6	7	7	7	6.25	6.70
DASH-SV Short Element 4 (debriefing)	56	0	6.61	0.68	5	6	7	7	7	6.40	6.76
DASH-SV Short Element 5 (debriefing)	56	0	6.23	1.06	4	5	7	7	7	5.93	6.49
DASH-SV Short Element 6 (debriefing)	56	0	6.52	0.81	4	6	7	7	7	6.27	6.70
DASH-SV Short introduction	56	0	6.75	0.61	5	7	7	7	7	6.55	6.88
DASH-SV Short debriefing	56	0	6.52	0.59	4.8	6.2	6.8	7	7	6.35	6.66
EPQ1	56	0	4.80	0.52	3	5	5	5	5	4.63	4.91
EPQ2	56	0	4.75	0.64	3	5	5	5	5	4.54	4.89
EPQ3	56	0	4.61	0.59	3	4	5	5	5	4.43	4.74
EPQ4	56	0	4.55	0.66	3	4	5	5	5	4.36	4.71
EPQ5	56	0	4.73	0.56	3	5	5	5	5	4.56	4.85

**Variable**	**N**	**Missing**	**M**	**SD**	**Min**	**Q1**	**Median**	**Q3**	**Max**	**Lower 95% CI**	**Upper 95% CI**
EPQ6	56	0	4.41	0.73	3	4	5	5	5	4.20	4.59
EPQ7	56	0	4.34	0.90	2	4	5	5	5	4.08	4.55
EPQ8	56	0	4.84	0.37	4	5	5	5	5	4.72	4.92
EPQ9	56	0	4.75	0.58	3	5	5	5	5	4.56	4.87
EPQ10	56	0	4.93	0.26	4	5	5	5	5	4.83	4.98
EPQ11	56	0	4.86	0.52	3	5	5	5	5	4.66	4.96
EPQ12	56	0	4.86	0.52	3	5	5	5	5	4.66	4.96
EPQ13	56	0	4.59	0.65	3	4	5	5	5	4.40	4.74
EPQ14	56	0	4.71	0.46	4	4	5	5	5	4.58	4.82
EPQ15	56	0	4.75	0.58	3	5	5	5	5	4.56	4.87
EPQ16	56	0	4.80	0.40	4	5	5	5	5	4.68	4.89
EPQ active learning	56	0	4.67	0.28	3.9	4.5	4.7	4.9	5	4.59	4.74
EPQ collaboration	56	0	4.86	0.52	3	5	5	5	5	4.66	4.96
EPQ diverse ways of learning	56	0	4.65	0.40	4	4.5	5	5	5	4.54	4.75
EPQ high expectations	56	0	4.78	0.41	3.5	4.5	5	5	5	4.65	4.87

Supplementary S2. Full Pairwise Correlation Matrix

Note. Pearson correlation coefficients are presented for all pairwise associations among study domains. Confidence intervals are reported at the 95% level. No multiplicity adjustment was applied; therefore, p values should be interpreted cautiously and alongside effect sizes and confidence intervals. Correlations do not imply causation.

Table S2. Full pairwise Pearson correlation matrix

Variable X	Variable Y	N	df	Pearson's r	95% CI	p
SSSCL satisfaction with learning	SSSCL self-confidence in learning	56	0.316	0.058	0.535	0.018
SSSCL satisfaction with learning	SDS objectives/information	56	0.299	0.039	0.521	0.025
SSSCL satisfaction with learning	SDS support	56	0.114	-0.153	0.366	0.402
SSSCL satisfaction with learning	SDS problem-solving	56	0.143	-0.124	0.392	0.292
SSSCL satisfaction with learning	SDS feedback/reflection	56	0.230	-0.035	0.465	0.088
SSSCL satisfaction with learning	SDS realism	56	0.088	-0.179	0.343	0.517
SSSCL satisfaction with learning	DASH-SV Short introduction	56	0.195	-0.071	0.436	0.149
SSSCL satisfaction with learning	DASH-SV Short debriefing	56	0.138	-0.130	0.387	0.311
SSSCL satisfaction with learning	EPQ active learning	56	0.355	0.102	0.565	0.007
SSSCL satisfaction with learning	EPQ collaboration	56	-0.047	-0.307	0.218	0.729
SSSCL satisfaction with learning	EPQ diverse ways of learning	56	-0.080	-0.336	0.187	0.560
SSSCL satisfaction with learning	EPQ high expectations	56	0.329	0.072	0.545	0.013
SSSCL self-confidence in learning	SDS objectives/information	56	0.227	-0.038	0.462	0.093
SSSCL self-confidence in learning	SDS support	56	0.238	-0.027	0.471	0.078
SSSCL self-confidence in learning	SDS problem-solving	56	0.122	-0.146	0.373	0.372
SSSCL self-confidence in learning	SDS feedback/reflection	56	-0.051	-0.310	0.215	0.707
SSSCL self-confidence in learning	SDS realism	56	0.178	-0.089	0.421	0.190
SSSCL self-confidence in learning	DASH-SV Short introduction	56	0.307	0.048	0.527	0.021
SSSCL self-confidence in learning	DASH-SV Short debriefing	56	0.303	0.043	0.524	0.023

Variable X	Variable Y	N	df	Pearson's r	95% CI	p
SSSCL self-confidence in learning	EPQ active learning	56	0.354	0.100	0.564	0.008
SSSCL self-confidence in learning	EPQ collaboration	56	0.209	-0.057	0.447	0.123
SSSCL self-confidence in learning	EPQ diverse ways of learning	56	0.083	-0.184	0.339	0.543
SSSCL self-confidence in learning	EPQ high expectations	56	0.268	0.005	0.496	0.046
SDS objectives/information	SDS support	56	0.633	0.444	0.768	< 0.001
SDS objectives/information	SDS problem-solving	56	0.704	0.541	0.816	< 0.001
SDS objectives/information	SDS feedback/reflection	56	0.582	0.377	0.733	< 0.001
SDS objectives/information	SDS realism	56	0.703	0.540	0.815	< 0.001
SDS objectives/information	DASH-SV Short introduction	56	0.179	-0.088	0.422	0.187
SDS objectives/information	DASH-SV Short debriefing	56	0.264	0.001	0.493	0.049
SDS objectives/information	EPQ active learning	56	0.202	-0.065	0.441	0.136
SDS objectives/information	EPQ collaboration	56	0.103	-0.164	0.356	0.450
SDS objectives/information	EPQ diverse ways of learning	56	-0.006	-0.268	0.258	0.968
SDS objectives/information	EPQ high expectations	56	0.158	-0.110	0.404	0.246
SDS support	SDS problem-solving	56	0.451	0.213	0.638	< 0.001
SDS support	SDS feedback/reflection	56	0.304	0.045	0.525	0.023
SDS support	SDS realism	56	0.904	0.840	0.943	< 0.001
SDS support	DASH-SV Short introduction	56	0.047	-0.219	0.306	0.733
SDS support	DASH-SV Short debriefing	56	0.127	-0.140	0.378	0.350
SDS support	EPQ active learning	56	0.263	0.000	0.492	0.050
SDS support	EPQ collaboration	56	0.174	-0.094	0.418	0.201
SDS support	EPQ diverse ways of learning	56	-0.003	-0.266	0.260	0.980
SDS support	EPQ high expectations	56	0.112	-0.155	0.364	0.411
SDS problem-solving	SDS feedback/reflection	56	0.685	0.514	0.803	< 0.001
SDS problem-solving	SDS realism	56	0.483	0.252	0.662	< 0.001

Variable X	Variable Y	N	df	Pearson's r	95% CI	p
SDS problem-solving	DASH-SV Short introduction	56	0.307	0.048	0.527	0.021
SDS problem-solving	DASH-SV Short debriefing	56	0.296	0.036	0.518	0.027
SDS problem-solving	EPQ active learning	56	0.106	-0.162	0.359	0.438
SDS problem-solving	EPQ collaboration	56	0.016	-0.248	0.278	0.905
SDS problem-solving	EPQ diverse ways of learning	56	0.072	-0.194	0.329	0.596
SDS problem-solving	EPQ high expectations	56	0.059	-0.208	0.317	0.668
SDS feedback/reflection	SDS realism	56	0.340	0.085	0.554	0.010
SDS feedback/reflection	DASH-SV Short introduction	56	0.128	-0.140	0.378	0.348
SDS feedback/reflection	DASH-SV Short debriefing	56	0.175	-0.092	0.418	0.198
SDS feedback/reflection	EPQ active learning	56	0.220	-0.046	0.456	0.104
SDS feedback/reflection	EPQ collaboration	56	-0.154	-0.401	0.113	0.256
SDS feedback/reflection	EPQ diverse ways of learning	56	0.108	-0.159	0.361	0.427
SDS feedback/reflection	EPQ high expectations	56	0.137	-0.131	0.386	0.315
SDS realism	DASH-SV Short introduction	56	0.093	-0.174	0.347	0.496
SDS realism	DASH-SV Short debriefing	56	0.169	-0.098	0.414	0.212
SDS realism	EPQ active learning	56	0.277	0.016	0.504	0.039
SDS realism	EPQ collaboration	56	0.254	-0.010	0.485	0.059
SDS realism	EPQ diverse ways of learning	56	-0.013	-0.275	0.251	0.926
SDS realism	EPQ high expectations	56	0.066	-0.200	0.324	0.627
DASH-SV Short introduction	DASH-SV Short debriefing	56	0.527	0.307	0.694	< 0.001
DASH-SV Short introduction	EPQ active learning	56	0.279	0.017	0.505	0.037
DASH-SV Short introduction	EPQ collaboration	56	0.229	-0.036	0.464	0.089
DASH-SV Short introduction	EPQ diverse ways of learning	56	0.120	-0.148	0.371	0.379
DASH-SV Short introduction	EPQ high expectations	56	0.171	-0.097	0.415	0.209
DASH-SV Short debriefing	EPQ active learning	56	0.311	0.052	0.531	0.020

Variable X	Variable Y	N	df	Pearson's r	95% CI	p
DASH-SV Short debriefing	EPQ collaboration	56	-0.084	-0.340	0.183	0.537
DASH-SV Short debriefing	EPQ diverse ways of learning	56	0.066	-0.200	0.323	0.629
DASH-SV Short debriefing	EPQ high expectations	56	0.268	0.005	0.496	0.046
EPQ active learning	EPQ collaboration	56	0.274	0.012	0.501	0.041
EPQ active learning	EPQ diverse ways of learning	56	0.478	0.246	0.658	< 0.001
EPQ active learning	EPQ high expectations	56	0.568	0.358	0.723	< 0.001
EPQ collaboration	EPQ diverse ways of learning	56	0.105	-0.162	0.358	0.440
EPQ collaboration	EPQ high expectations	56	0.102	-0.165	0.356	0.453
EPQ diverse ways of learning	EPQ high expectations	56	0.478	0.246	0.658	< 0.001

Supplementary S3. TREND Checklist

This checklist follows the Transparent Reporting of Evaluations with Nonrandomized Designs (TREND Statement - Des Jarlais et al., 2004) and the Reporting Guidelines for Healthcare Simulation Research (Cheng et al., 2016). Items that are not applicable because this study used a single-group educational intervention without a comparison group are identified accordingly.

Table S3. TREND checklist

Item	Topic	TREND recommendation	Manuscript location	Status / notes
Title and Abstract	Title and Abstract	Identify allocation method and study design	Title; Abstract	Reported as a single-group educational intervention study.
Background	Scientific background	Explain rationale and theoretical basis	Introduction	Reported.
Objectives	Objectives	State specific objectives and hypotheses	End of Introduction	Reported. Exploratory objectives stated.
Participants	Eligibility criteria	Eligibility criteria, recruitment methods, setting	Methods 2.2	Reported.
Participants	Recruitment	Recruitment procedures	Methods 2.2	Reported. All students enrolled in the course were invited.
Intervention	Intervention	Describe intervention in sufficient detail	Methods 2.3–2.4	Reported. Standardized simulation, prebriefing, scenario and debriefing described.
Intervention	Intervention delivery	Who delivered intervention, how, where and when	Methods 2.3–2.4	Reported.
Outcomes	Outcome measures	Clearly define primary and secondary outcomes	Methods 2.5	Reported. Learner-reported outcomes using validated instruments.
Sample size	Sample size	Explain determination of sample size	Methods 2.2	Reported. Convenience sample; no a priori calculation because course-embedded exploratory study and the study was not designed as a confirmatory effectiveness trial.
Assignment method	Allocation	Method used to assign participants	Not applicable	Single-group educational intervention; no allocation or comparison group.
Blinding	Blinding	Whether participants or assessors were blinded	Methods 2.8	Not applicable. Educational intervention; anonymous questionnaires minimized response bias.
Unit of analysis	Unit of analysis	Describe unit analyzed	Methods 2.7	Individual student.
Statistical methods	Statistical analysis	Statistical methods used	Methods 2.7	Reported. Descriptive statistics, exploratory Pearson correlations, Benjamini–Hochberg FDR adjustment for prespecified correlations.

Item	Topic	TREND recommendation	Manuscript location	Status / notes
Participant flow	Flow	Flow through each stage	Results 3.1	Reported. All 56 students completed questionnaires; no missing data.
Baseline data	Baseline characteristics	Baseline demographic characteristics	Results 3.1; Table 1	Reported.
Numbers analyzed	Analysis	Numbers included in each analysis	Results 3	Reported. Complete-case analysis.
Outcomes	Results	Summary measures and effect estimates	Results 3.2–3.3	Reported. Means, SDs, 95% CIs, Pearson r and adjusted p-values.
Ancillary analyses	Additional analyses	Additional analyses performed	Results 3.3; Supplementary Appendix B	Reported as exploratory correlations.
Adverse events	Harms	Adverse events or unintended effects	Results	None observed during the educational activity.
Interpretation	Interpretation	Interpretation considering limitations	Discussion	Reported. Exploratory findings interpreted cautiously.
Generalizability	External validity	Discuss generalizability	Discussion 4.2	Reported. Single institution and single cohort.
Overall evidence	Interpretation	Place findings in context	Discussion; Conclusions	Reported.
Ethics	Ethics	Ethical approval and informed consent	Methods 2.8	Reported. Consent referred to participation in the research questionnaires; simulation activity was part of the curriculum.

Note. Several TREND items related to treatment allocation, comparison groups, and participant assignment are not applicable because this study evaluated a standardized educational intervention using a single-group post-intervention design embedded within the undergraduate curriculum.

Supplementary S4. Simulation Scenario Script and Observer Checklist

This appendix provides a de-identified scenario outline and observer checklist for the leadership-focused high-fidelity simulation. Local occupational-health procedures and institutional protocols should be adapted before educational reuse.

Table S4. Scenario overview

Element	Description
Scenario title	Accident with a Sharp Object: Leadership Response to a Sharps-Injury Event
Target learners	Fourth-year undergraduate nursing students enrolled in a leadership/administration-related nursing course.
Learning focus	Leadership behaviors during an occupational-exposure event: communication, prioritization, delegation, emotional support, team coordination, decision-making, and reflective practice.
Estimated duration	50 minutes total: 5 minutes prebriefing/familiarization, 15 minutes scenario enactment, 30 minutes debriefing.
Setting	Hospital unit or simulated clinical room configured as a patient-care environment.
Fidelity level	High-fidelity adult patient simulator plus trained actors/standardized participants.
Patient background	Adult inpatient receiving routine nursing care. The patient is clinically stable but anxious after witnessing a staff member sustain a sharps injury.
Trigger event	A staff member sustains a needlestick/sharps injury while performing or assisting with a clinical procedure.
Expected leadership actions	Recognize exposure risk; stop unsafe activity; ensure immediate first aid; support the injured professional; maintain patient safety; delegate tasks; notify appropriate services; follow institutional occupational-health protocol; communicate clearly with the team; document or verbalize required follow-up steps.
Scenario end point	Learner has coordinated immediate safety response, supported the injured professional, stabilized the clinical environment, and initiated escalation/follow-up according to local protocol.

Table S5. Suggested roles and scripted cues

Role	Scripted function / cue
Learner nurse leader / charge nurse	Expected to coordinate the response, communicate priorities, delegate tasks, and initiate institutional procedures.
Injured staff member / standardized participant	Displays anxiety and uncertainty; reports sharps injury; asks whether they are at risk and what will happen next.
Assisting staff member	Can follow delegated instructions; may ask for clarification if instructions are vague.
Patient simulator / patient voice	Stable but anxious; may ask whether the incident affects their care.
Facilitator	Maintains scenario flow, provides cues only if learners are stuck, protects psychological safety, and observes leadership behaviors.
Observers	Use the observer checklist to identify leadership behaviors and prepare for debriefing participation.

Table S6. Scenario progression

Phase	Expected event and learner focus
Prebriefing	Facilitator establishes psychological safety, confidentiality, fiction contract, role expectations, observer task, learning objectives, and non-punitive learning orientation.

Phase	Expected event and learner focus
Minute 0-3	Sharps injury occurs; injured staff member verbalizes distress. Expected learner behavior: pause activity, ensure immediate safety, acknowledge injury, organize response.
Minute 3-7	Team uncertainty increases. Expected learner behavior: delegate first aid, patient reassurance, notification, and protocol steps.
Minute 7-12	Facilitator or actor asks about next steps. Expected learner behavior: verbalize occupational-health follow-up, documentation/escalation, emotional support, and team communication.
Minute 12-15	Scenario closes once immediate response is coordinated and follow-up is initiated.
Debriefing	Analyze leadership behaviors, communication clarity, prioritization, emotional support, role allocation, decision-making, and transfer to clinical practice.

Table S7. Observer checklist for leadership behaviors

Domain	Observable behavior	Not observed	Partially observed	Clearly observed	Comments
Recognition and prioritization	Recognizes the sharps injury promptly and treats it as a safety event.	☐	☐	☐	
Recognition and prioritization	Stops or redirects unsafe activity and protects patient/team safety.	☐	☐	☐	
Recognition and prioritization	Prioritizes immediate first aid and exposure-management steps.	☐	☐	☐	
Communication	Uses clear, calm, and direct communication with the injured professional.	☐	☐	☐	
Communication	Communicates role expectations to team members.	☐	☐	☐	
Communication	Keeps the patient informed without increasing anxiety.	☐	☐	☐	
Delegation and coordination	Delegates tasks appropriately to available team members.	☐	☐	☐	
Delegation and coordination	Checks whether delegated tasks were understood or completed.	☐	☐	☐	
Delegation and coordination	Coordinates escalation or notification according to local policy.	☐	☐	☐	
Emotional support	Acknowledges emotional distress of the injured professional.	☐	☐	☐	
Emotional support	Provides reassurance while avoiding false promises.	☐	☐	☐	

Domain	Observable behavior	Not observed	Partially observed	Clearly observed	Comments
Problem-solving	Identifies required resources or next steps under time pressure.	☐	☐	☐	
Problem-solving	Adapts actions when new information or uncertainty emerges.	☐	☐	☐	
Professional practice	Verbalizes or initiates documentation/follow-up requirements.	☐	☐	☐	
Professional practice	Demonstrates professional integrity and non-punitive response.	☐	☐	☐	
Teamwork	Encourages participation and shared situational awareness.	☐	☐	☐	
Teamwork	Maintains team focus without dismissing concerns.	☐	☐	☐	
Reflection prompt	Identifies one leadership strength for debriefing.	☐	☐	☐	
Reflection prompt	Identifies one leadership improvement point for debriefing.	☐	☐	☐	

Note. The observer checklist is designed for observer learning and debriefing support, not for high-stakes competency assessment unless formally validated for that purpose.

Supplementary S5. Instrument, Scoring, and Permission Documentation Notes

This appendix documents the instruments, scoring approach, and permission considerations relevant to the supplementary materials. It does not reproduce copyrighted instrument items. Authors should retain the original instrument packets, permissions, translation/adaptation documentation, and scoring manuals in the study file.

E1. Missing-data and domain-score documentation

No missing data were identified in the dataset. Therefore, all descriptive statistics and Pearson correlation analyses were conducted using complete-case data, with a constant sample size across analyses. No item imputation procedures were required. The exact sample size (N) for each analysis is reported in Supplementary Appendices A and B.

Table S8. Instrument and scoring documentation

Instrument / material	Domains or elements used	Response scale / scoring used in manuscript	Documentation / permission note
Simulation Design Scale (SDS)	Objectives/information; support; problem-solving; feedback/reflection; realism.	Items and domains analyzed as means on a 1-5 scale. Higher scores indicate more favorable perceptions. If the instrument version included parallel importance ratings, only the evaluation/presence stream was used for the reported analyses.	Use the authorized NLN/Laerdal instrument version or the validated/adapted version available to the research team. Do not reproduce full items without permission. Insert exact Brazilian adaptation/validation citation if applicable.
Student Satisfaction and Self-Confidence in Learning Scale (SSSCL)	Satisfaction with learning; self-confidence in learning.	Items and subscales analyzed as means on a 1-5 scale. Higher scores indicate greater satisfaction or self-confidence.	Use the authorized instrument version and exact translation/adaptation citation. Standardize acronym as SSSCL throughout manuscript and supplement.
Educational Practices Questionnaire (EPQ)	Active learning; collaboration; diverse ways of learning; high expectations.	Items and domain scores analyzed as means on a 1-5 scale. Higher scores indicate more favorable perceptions of educational practices.	Verify that the administered version uses this domain name; do not reproduce full items without permission. Insert exact adaptation/validation citation if applicable.
Debriefing Assessment for Simulation in Healthcare - Student Version, Short Form (DASH-SV Short)	Element 1: introduction; Elements 2-6: debriefing quality.	Elements rated on a 1-7 scale and analyzed as element/domain means. Higher scores indicate more favorable student ratings of introduction and debriefing.	Use the official Center for Medical Simulation DASH documentation and retain permission documentation in the study file. Do not reproduce copyrighted DASH items unless permission allows reproduction.
Observer checklist	Leadership behaviors: recognition/prioritization, communication, delegation, emotional support, problem-solving, professional practice, teamwork, reflection prompts.	Used as an educational observation and debriefing support tool, not as a validated high-stakes performance assessment.	Original educational checklist developed for the scenario. It may be included as supplementary material because it is not a proprietary instrument.
Simulation scenario script	Sharps-injury event requiring leadership response.	Scenario progression and expected leadership behaviors documented for reproducibility.	Adapt local occupational-health protocols before reuse. If local policy details are institution-specific, de-identify for blinded review.

E2. Permission and copyright notes for submission

The instruments used in this study (DASH-SV Short, Educational Practices Questionnaire, Simulation Design Scale, and Student Satisfaction and Self-Confidence in Learning Scale) were obtained from versions published in the scientific literature and from previously published translation, cultural adaptation, and validation studies. All original sources and validated versions used by the research team have been appropriately cited in the manuscript.

The authors do not claim copyright ownership of these instruments. In accordance with applicable copyright requirements, complete copies of copyrighted instruments will not be provided without specific authorization from the respective copyright holders. If requested, bibliographic references and information regarding the versions used in this study will be provided.

The official citation for the DASH-SV Short has been retained and cited according to the original source.

The simulation scenario script and observer checklist included in Supplementary Appendix D are original educational materials developed by the authors, have been de-identified, and may be submitted as supplementary material.

Table S9. Source and permission links to retain in the study file

Table S9. Source and permission links to retain in the study file

Resource	Official webpage / URL
STROBE cross-sectional checklist	https://www.strobe-statement.org/checklists/
Clinical Simulation in Nursing Guide for Authors	https://www.sciencedirect.com/journal/clinical-simulation-in-nursing/publish/guide-for-authors
INACSL Healthcare Simulation Standards of Best Practice	https://www.inacsl.org/healthcare-simulation-standards
Center for Medical Simulation – DASH-SV Short	https://harvardmedsim.org/debriefing-assessment-for-simulation-in-healthcare-dash/
Center for Medical Simulation DASH Portuguese information page	https://harvardmedsim.org/debriefing-assessment-for-simulation-in-healthcare-dash-portuguese/
DASH Student Version Short Form PDF	https://harvardmedsim.org/wp-content/uploads/2017/01/DASH.SV.Short.2010.Final.pdf
Portuguese cross-cultural adaptation of DASH	Couto TB, Kerbe M, Mollo V, et al. (2021). *Translation of the Debriefing Assessment for Simulation in Healthcare in Portuguese and cross-cultural adaptation for Portugal and Brazil*. Advances in Simulation, 6, 25. https://doi.org/10.1186/s41077-021-00175-z
National League for Nursing (NLN) Tools and Instruments	https://www.nln.org/education/teaching-resources/tools-and-instruments
Student Satisfaction and Self-Confidence in Learning Scale (SCLS)	https://www.nln.org/docs/default-source/uploadedfiles/default-document-library/instrument-2-satisfaction-and-self-confidence-in-learning.pdf
Portuguese validation of Student Satisfaction and Self-Confidence in Learning Scale	Almeida RGS, Mazzo A, Martins JCA, Baptista RCN, Girão FB, Mendes IAC. (2015). *Validation to Portuguese of the Scale of Student Satisfaction and Self-Confidence in Learning*. Revista Latino-Americana de Enfermagem, 23(6), 1007–1013. https://doi.org/10.1590/0104-1169.0472.2643
Portuguese validation of Simulation Design Scale (SDS)	Almeida RGS, Mazzo A, Martins JCA, Pedersoli CE, Fumincelli L, Mendes IAC. (2015). *Validação para a língua portuguesa da Simulation Design Scale*. Texto & Contexto Enfermagem, 24(4), 934–940. https://doi.org/10.1590/0104-07072015000004570014

Resource	Official webpage / URL
Portuguese validation of Educational Practices Questionnaire (EPQ – Student Version)	Almeida RGS, Mazzo A, Martins JCA, Souza Júnior VD, Mendes IAC. (2016). *Validação para a língua portuguesa do Educational Practices Questionnaire (Student Version)*. Acta Paulista de Enfermagem, 29(4), 390–396. https://doi.org/10.1590/1982-0194201600054

References for Supplementary Materials

Brett-Fleegler, M., Rudolph, J., Eppich, W., Monuteaux, M., Fleegler, E., Cheng, A., & Simon, R. (2012). Debriefing Assessment for Simulation in Healthcare: Development and psychometric properties. Simulation in Healthcare, 7(5), 288-294. https://doi.org/10.1097/SIH.0b013e3182620228

Center for Medical Simulation. (2010). Debriefing Assessment for Simulation in Healthcare (DASH): Student Version, Short Form. https://harvardmedsim.org/wp-content/uploads/2017/01/DASH.SV.Short.2010.Final.pdf

Cheng, A., Kessler, D., Mackinnon, R., Chang, T. P., Nadkarni, V. M., Hunt, E. A., Duval-Arnould, J., Lin, Y., Cook, D. A., Pusic, M., Hui, J., Moher, D., Egger, M., & Auerbach, M. (2016). Reporting guidelines for health care simulation research: Extensions to the CONSORT and STROBE statements. Simulation in Healthcare, 11(4), 238-248. https://doi.org/10.1097/SIH.0000000000000150

Clinical Simulation in Nursing. (2026). Guide for authors. ScienceDirect. Retrieved June 8, 2026, from https://www.sciencedirect.com/journal/clinical-simulation-in-nursing/publish/guide-for-authors

Decker, S., Sapp, A., Bibin, L., Chidume, T., Crawford, S. B., Fayyaz, J., Johnson, B. K., & Szydlowski, J. (2025). Healthcare Simulation Standards of Best Practice: The debriefing process. Clinical Simulation in Nursing, 105, Article 101775. https://doi.org/10.1016/j.ecns.2025.101775

Persico, L., Ramakrishnan, S., Wilson-Keates, B., Catena, R., Charnetski, M., Fogg, N., Jones, M. C., Ludlow, J., MacLean, H., Simmons, V. C., Smeltzer, S., & Wilk, A. (2025). Healthcare Simulation Standard of Best Practice: Prebriefing preparation and briefing. Clinical Simulation in Nursing, 105, Article 101777. https://doi.org/10.1016/j.ecns.2025.101777

STROBE Statement. (2026). STROBE checklists. Retrieved June 8, 2026, from https://www.strobe-statement.org/checklists/

Watts, P. I., McDermott, D. S., Alinier, G., Charnetski, M., Ludlow, J., Horsley, E., Meakim, C., & Nawathe, P. (2021). Healthcare Simulation Standards of Best Practice: Simulation design. Clinical Simulation in Nursing, 58, 14-21. https://doi.org/10.1016/j.ecns.2021.08.009
